# Research progress of airway inflammation in asthma: A bibliometric analysis

**DOI:** 10.1097/MD.0000000000038842

**Published:** 2024-07-19

**Authors:** Lang Liye, Zhao Hui, Huang Fuchun, Liu Hua

**Affiliations:** a Department of Respiratory and Critical Care Medicine, Affiliated Hospital of Nantong University, Medical School of Nantong University, Nantong, China.

**Keywords:** airway inflammation, asthma, bibliometric analysis, visualization analysis, Web of Science

## Abstract

**Background:**

In recent years, the prevalence of asthma has gradually increased and the number of asthmatics worldwide has reached 358 million, which has caused huge economic loss. Airway inflammation is an important feature of asthma, and international research in this field has a high degree of heat. Therefore, this paper uses the bibliometric method to systematically review and visualize the literature in this field, aiming to provide some reference value for follow-up related research.

**Methods:**

To retrieve the research literature on airway inflammation in asthma from 2003 to 2022 in the Web of Science Core Collection database. The bibliometric method was used to systematically analyze the included literature data by using visualization analysis software such as CiteSpace (6.2. R4) and VOSviewer (1.6.19).

**Results:**

A total of 1892 articles published in 423 journals were included in this study, from 1912 institutions in 62 countries/regions. The number of articles published between 2003 and 2022 showed a trend of fluctuating growth. The country with the largest number of articles published was China (558,29.49 %), followed by the United States (371,19.61 %) and Korea (212,11.21 %). Gibson, Peter G is the author with the highest number of publications, and Journal of Allergy and Clinical Immunology is the most published journal.

**Conclusion subsections:**

This study systematically reveals the state of the literature in the field of airway inflammation in asthma over the past 20 years. The exploration of inflammatory cell components, pathway molecules and biological agents are research hotspots in this field and should be further studied.

## 1. Introduction

Asthma is a chronic airway inflammatory disease involving a variety of cells and cellular components. The clinical manifestations are recurrent wheezing, shortness of breath, with or without chest tightness or cough, accompanied by airway hyperresponsiveness and variable airflow limitation.^[1]^ Asthma affects the patient daily work and study, resulting in a decline in the patient health and quality of life.^[2,3]^ It is often difficult to get effective control treatment because of repeated asthma attacks, high costs of control treatment and long-team treatment.^[4]^ Over the years, the global prevalence and mortality of asthma have been at a high level,^[5]^ resulting in a significant social and economic burden,^[6]^ which has forced researchers to conduct more in-depth research on asthma. As one of the key features of asthma, airway inflammation is closely related to asthma and is the core link of its pathogenesis.^[7–9]^ Therefore, it is very important to grasp the research status and hot trends of asthmatic airway inflammation for further research in this field. Although a large number of studies have conducted in-depth research and review on asthmatic airway inflammation, there is no relevant document to systematically analyze and compare this field, which makes it difficult for researchers to fully understand the current challenges and gaps in the field of airway inflammation. Bibliometrics is a discipline that takes the literature characteristics as the research object, uses statistical methods to study and summarize the distribution law of literature in specific fields.^[10–14]^ Bibliometrics can help researchers to find the development keywords in specific fields and show the evolution process of research fields through the visualization of data map.^[15–19]^ Therefore, this paper will adopt the bibliometric method to visually analyze and display the literature data related to asthmatic airway inflammation included in the Web of Science Core Collection (WOSCC) database, in order to provide more new ideas for research in this field.

## 2. Materials and methods

### 2.1. Data source and research strategies

We selected the WOSCC database as the data source, and the retrieval time span was from 2003-01-01 to 2022-12-31. The search conditions were as follows: TS = (asthma* OR “bronchial asthma” OR wheeze OR wheezing OR “shortness of breath” OR “bronchial spasm” OR bronchospasm OR “bronchial hyperreactivity” OR “bronchial hyperresponsiveness”) AND TS = (“airway inflammation” OR “bronchial inflammation” OR “airway inflammatory” OR “respiratory tract inflammation”). All searches were completed and downloaded on October 8,2023 in case of bias caused by database updates.

### 2.2. Literature screening and data extraction

**Figure 1.**
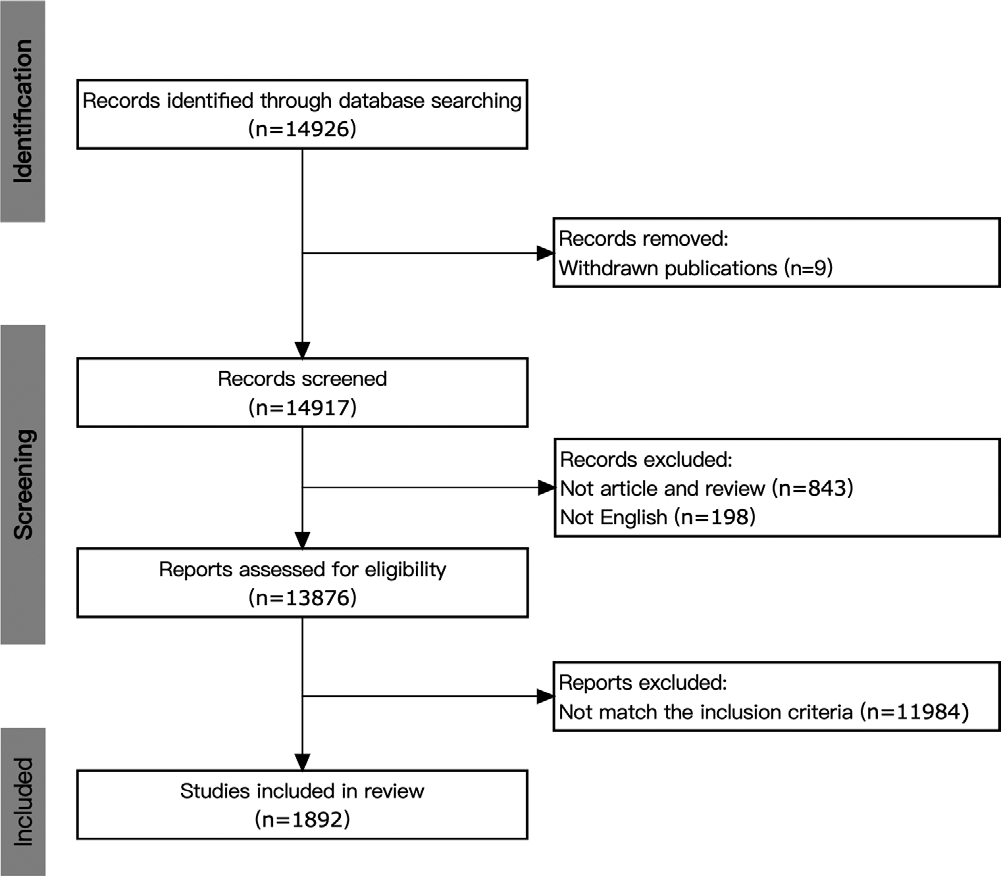
Screening flow chart.

A total of 14926 articles were initially retrieved, and selected according to the following criteria: literature type: paper or review paper; language: English. Initially, there were 13,876 articles met these criteria. After manually screening the titles and abstracts of these articles, 1892 articles specifically on this topic were finally selected. The selection process of articles is presented in Figure 1.

### 2.3. Data analysis

We used CiteSpace (6.2.R4) and VOSviewer (1.6.19) to visually analyze the time, author, country, institution, journal, keywords and cited references of the literature related to asthmatic airway inflammation, and used GraphPad Prism (9.5.1) to draw clear pictures. By analyzing and demonstrating the layout of asthmatic airway inflammation, the research status is described, so as to predict the research hotspots and trends in this field.

### 2.4. Data quality control

In order to ensure the authenticity and accuracy of the research results, data extraction was performed independently by 2 researchers, and the titles, abstracts and keywords of the articles were screened according to the inclusion and exclusion criteria. In case of disagreement in the process, a third party with a professional background is asked to negotiate and judge.

## 3. Results

### 3.1. Annual publication trend

**Figure 2.**
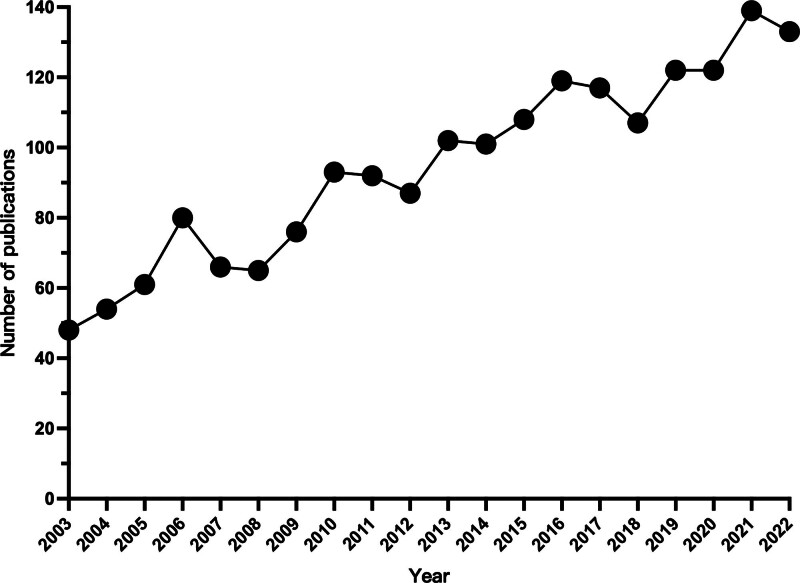
Trends in annual publication growth from 2003 to 2022.

There were 1756 articles (92.81%) and 136 reviews (7.19%) among the 1892 documents. It can be seen from Figure 2 that the annual number of publications on asthmatic airway inflammation between 2003 and 2022 showed a fluctuating upward trend. From 2003 to 2009, annual publication volume was still low, with an average annual publication of 64.3. After 2009, the annual number of publications began to increase gradually. As of 2022, the average annual number of publications was as high as 110.9, with the highest number of publications in 2021, accounting for 7.35 % (139/1892). This shows that more and more scholars have begun to pay attention to the study of airway inflammation in asthma worldwide.

### 3.2. Author analysis

**Figure 3.**
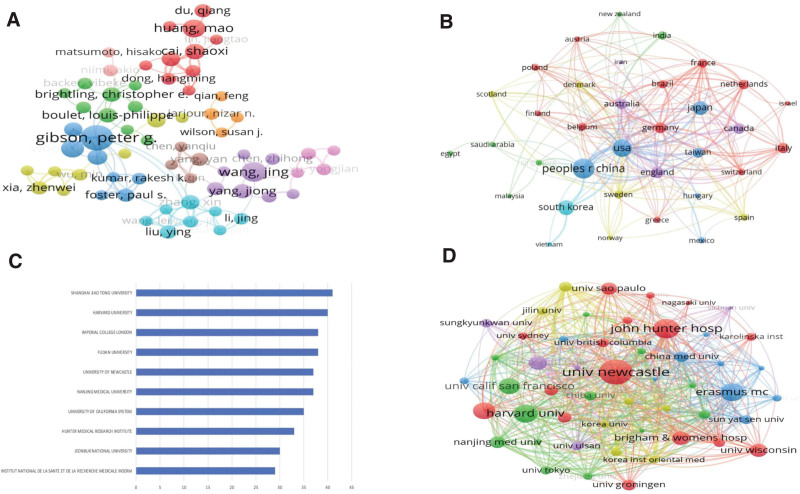
(A) The Visualization network diagram of collaborations between core authors. Different colors represent different clusters. The size of the node indicates the number of published papers. The thickness of the line indicates the intensity of cooperation between authors. (B) Visualization map of cooperation between countries with more than 5 articles. Each node represents a country/ region. The size of the node represents the number of posts. The thickness of the connection indicates the intensity of cooperation between countries. (C) The top 10 institutions with the most publications. (D) The visualization map of collaborations between the top 50 institutions. One node represents an institution. The size of the node indicates the number of article references. The connection between nodes indicates the cooperation between institutions.

The top 10 authors were mainly from Asia and Australia (Table 1). The top 3 productive authors are Gibson, Peter G (n = 29), Shin, I (n = 24) and Dong, Jingcheng (n = 17). Among them, Gibson, Peter G from Australia is ranked first in the total citation with 2726 citations. The average citation of Wood, Lisa G (108.33) is the highest among the top 10 authors, indicating that Gibson, Peter G and Wood, Lisa G have important influence in this field. Pryce law can help us understand the distribution of authors in the research field and identify the core authors with important influence. By calculating the formula, it can be drawn the conclusion that the authors who have published more than 4 papers are considered to be the core authors in this field. Figure 3A shows 301 core authors with ≥ 4 articles, forming 10 clusters. It can be seen that the research team with Gibson, Peter G as the core has a close cooperation with other authors, followed by Zhang, xin team in this field with other researchers.

**Table 1:** The top 10 authors with the most publications.

Author	Country/Region	Articles	Citations	Average citation
Gibson, Peter G	Australia	29	2726	94
Shin, I	Korea	24	510	21.25
Dong, Jingcheng	China	17	352	20.71
Lee, Mee-Young	Korea	16	375	23.44
Wood, Lisa G	Australia	15	1625	108.33
Oh, Sei-Ryang	Korea	14	394	28.14
Boulet, Louis-Philippe	Canada	14	640	45.71
Chiang, Bor-Luen	Taiwan of China	13	323	24.85
Yan, Guanghai	China	13	281	21.62
Mabalirajan, Ulaganathan	India	12	389	32.42

### 3.3. Country/Region analysis

The 1892 articles included in the study were from 62 countries/Regions. As shown in Table 2, the top 3 countries in terms of the number of publications were China (n = 558, 29.50%), the United States (n = 371, 19.61%) and Korea (n = 212, 11.21%). The total citations of articles in the United States ranks first, and its average citation also ranks in the top 3. The average citation of articles in the England is higher than that of other countries, followed by Australia. This shows that the United States, England and Australia have a strong influence in the field of asthmatic airway inflammation. In the national cooperation network map with more than 5 articles (Fig. 3B), it can be seen that the United States has close cooperation with many countries, among which the cooperation with China, England, Germany and Canada is the most obvious. Other countries that cooperate with each other are also mostly concentrated in western countries such as the United States, England, Australia, Germany and France, which suggests that European and American countries occupy a central leading position in the field of asthmatic airway inflammation research.

**Table 2:** The top 10 countries/regions with the most publications.

Rank	Country/region	Articles	Citations	Average citation
1	China	558	9852	17.66
2	The United States	371	18925	51.01
3	Korea	212	5972	28.17
4	Japan	179	6554	36.61
5	England	103	6487	62.98
6	Germany	87	4310	49.54
7	Canada	81	3684	45.48
8	Australia	77	4276	55.53
9	Italy	69	3409	49.41
10	Taiwan of China	64	1495	23.36

### 3.4. Institution analysis

The 1892 articles included in this study were from 1912 institutions, of which the top 10 institutions contributed 18.92 % (358/1892) of the articles. Figure 3C shows that the top 3 institutions were Shanghai Jiao Tong University (n = 41), Harvard University (n = 40) and Fudan University (n = 38). There are 8 university institutions in the top 10 institutions, which indicates that the research in the field of asthmatic airway inflammation is mainly based on university institutions. It can be seen from the top 50 institutions cooperation network map (Fig. 3D) that the cooperation between institutions is frequent, among which the University of Newcastle in Australia occupies a relatively dominant position, and the cooperation with John Hunt Hospital is the closest.

### 3.5. Journal analysis

**Figure 4.**
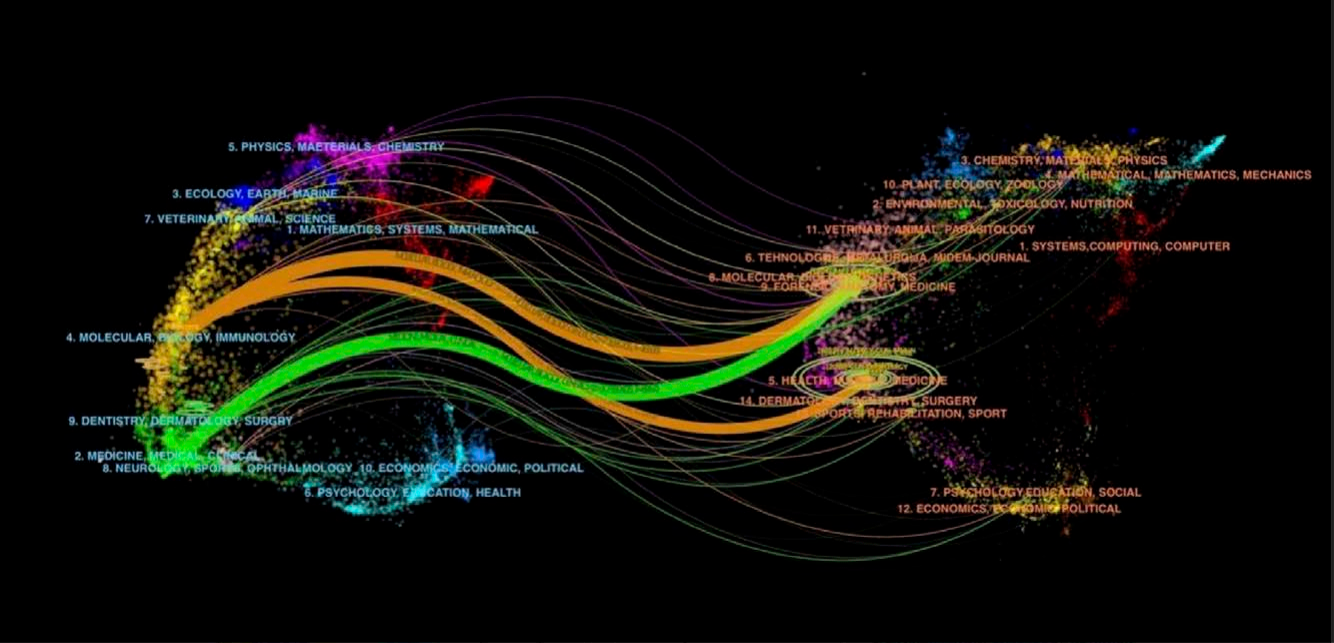
A dual-map overlay of journals related to research on asthmatic airway inflammation.

A total of 423 journals published articles on airway inflammation in asthma, of which 91 journals published more than 5 articles. Table 3 lists the top 10 journals in the number of published articles, and these journals accounted for 27.43 % (519/1892) of the total number of documents. *Journal of Allergy and Clinical Immunology* is ranked first with 89 articles, followed by *Clinical and Experimental Allergy* (n = 76). The total citations (5544) and average citation (62.29) of *Journal of Allergy and Clinical Immunology* (IF = 14.2) were ranked first and second respectively. This shows that it has important influence in this field. The scientific distribution of periodicals can be seen through the double superposition graph of journals (Fig. 4). The citing journal is on the left side, and the cited journal is on the right side. The color path illustrates the citation relationship, indicating the citation trajectory and knowledge flow.^[20]^ It can be found that the citing papers are mainly concentrated on the molecular, biological, immune, pharmaceutical and clinical fields, while the cited papers are mainly from the journals related to health, nursing, medicine, molecular, biological and genetic fields.

**Table 3:** The top 10 journals.

Source	Articles	Citations	Average citation	IF (2022)	JCR (2022)	Country
Journal of Allergy and Clinical Immunology	89	5544	62.29	14.2	Q1	The United States
Clinical and Experimental Allergy	76	3653	48.07	6.1	Q1	England
International Immunology	62	1583	25.53	5.6	Q1	England
Journal of Immunology	55	3514	63.89	4.4	Q2	The United States
PloS One	51	1134	22.24	3.7	Q2	The United States
International Archives of Allergy and Immunology	48	1010	21.04	2.8	Q3	Switzerland
Respiratory Research	37	1161	31.38	5.8	Q1	England
Allergy	36	1269	35.25	12.4	Q1	England
Journal of Asthma	35	576	16.46	1.9	Q4	The United States
Frontiers in Immunology	30	587	19.57	7.3	Q1	Switzerland

### 3.6. Keyword analysis

#### 3.6.1. Keyword co-occurrence

**Figure 5.**
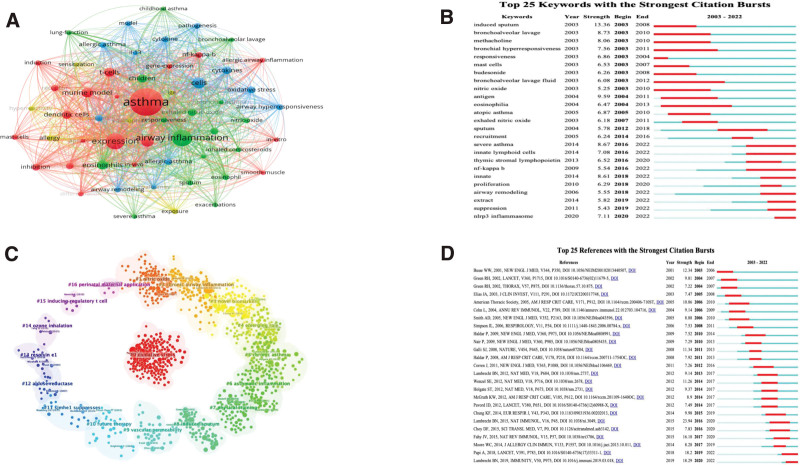
(A) Keyword co-occurrence network of asthmatic airway inflammation. (B) Keyword burst map of asthma airway inflammation. (C) Clustering of the co-cited references in asthma airway inflammation. (D) Citation burst map in the field of asthma airway inflammation.

VOSviewer software is used to generate the keyword co-occurrence network map of the literature (Fig. 5A). Among the 6296 keywords, the number of co-occurrences was set to ≥ 50 times, and 63 keywords were finally included in the analysis. By observing the co-occurrence network map, it can be found that the words “asthma,” “airway inflammation,” “expression,” “mouse model,” “activation,” “T cells,” “eosinophils,” “children,” “dendritic cells,” “cytokines,” and “oxidative stress” appear more frequently in these literatures.

#### 3.6.2. Keyword burst

The burst keywords can reflect the frontier trend of the field to a certain extent. As shown in Figure 5B, 25 keywords with high burst strength were screened. It can be seen that the keywords of early research include “induced sputum,” “bronchoalveolar lavage,” “methacholine,” “mast cells,” “nitric oxide” and “allergic asthma.” The keywords such as “severe asthma,” “innate lymphoid cells,” “thymic stromal lymphopoietin,” “NF-κB(nuclear factor-κ-gene binding),” “airway remodeling” and “NLRP3(nucleotide-binding oligomerization domain-like receptor protein 3) inflammasome” have appeared in recent years and continue to 2022. It is worth noting that “severe asthma,” “NLRP3 inflammasome,” “innate lymphoid cells” and “NF-κB” are the hot keywords with high intensity and long duration.

### 3.7. Reference analysis

#### 3.7.1. Clustering of co-cited references

CiteSpace software is used to generate a cluster diagram of references (Fig. 5C), so that we can better extract relevant references for research. In this co-cited reference cluster diagram, 17 clusters with specific significance can be found, including “# 0 oxidative stress,” “# 1 nitric oxide,” “# 2 chronic airway inflammation” and “# 3 new biomarkers.”

#### 3.7.2. Cited reference burst

Citation bursts can be used to indicate whether the article has attracted the attention of the scientific community. By observing the citation burst map (Fig. 5D), the first few references with the strongest citation burst since 2003 are: “The immunology of asthma,” “The Cytokines of Asthma,” “Asthma” and “Type 2 inflammation in asthma-present in most, absent in many.”

## 4. Discussion

### 4.1. Current research status

This study used CiteSpace and VOSviewer to conduct bibliometric and visualization analysis of 1892 publications related to asthmatic airway inflammation published in the WOSCC database from 2003 to 2022, and systematically sorted out the development status, hot trends and cooperation context of this research field. From 2003 to 2022, publications related to asthmatic airway inflammation showed a trend of fluctuating growth. Between 2003 and 2009, the number of publications related to airway inflammation in asthma was still low. Since 2010, the number of publications has remained at a high level, especially since 2013, the number of publications has exceeded 100 papers per year. These results suggest that between 2013 and 2022, research on airway inflammation in asthma has received increasing attention.

Among the top 10 authors in the number of published papers, Gibson, Peter G and Wood, LISA G have high total citation frequency and average citation frequency, and have published many articles in international authoritative journals such as *European Journal of Respiratory Medicine* and *Journal of Allergy and Clinical Immunology*. An article published by Wood, Lisa G et al in 2003 showed that oxidative stress is significant to the development of airway inflammation. Further study on the mechanism of oxidative stress and antioxidant defense system in airway inflammation is helpful to develop new treatment strategies and improve the prognosis of respiratory diseases such as asthma.^[21]^

In addition, Wood, Lisa G et al pointed out in another article that high fat intake in obese patients will increase airway inflammation in asthma.^[22]^ For overweight and obese asthmatics, planning weight loss goals and changing dietary fat intake are helpful for clinical management and treatment.^[23–26]^ In 2014, Gibson, Peter G et al found that the expression of NLRP3 inflammasome was increased in neutrophilic asthma, and further proved that NLRP3 and Caspase-1 proteins were present in sputum macrophages and neutrophils of neutrophilic respiratory asthma.^[27]^ The number of publications in a country can be used to assess its academic level.^[28]^ Table 2 shows that China, the United States, Korea, Japan, and England are the top 5 countries and account for 76.22% of the total number of papers, which suggests that these countries have more in-depth research in asthmatic airway inflammation. It is worth noting that the total number of articles, the total cited frequency and the average cited times of the United States are all in the forefront, and the total number of citations of the articles is far more than that of other countries. These results suggest that the United States has great influence and a leading position in this field. It can be found from Figure 3B that he United States, England and Germany work most closely with other countries/ regions. Therefore, the United States, England and Germany play an important role in the pattern of international cooperation, and they have established extensive cooperation. Analyzing the characteristics of international journals helps to identity and select important journals in the field and those suitable for publication. As shown in Table 3, *Journal of Allergy and Clinical Immunology*, *Clinical and Experimental Allergy*, *International Immunology, Respiratory Research*, *Allergy* and *Frontiers in Immunology* are not only the top 10 journals with the most published papers, but also the JCR Q1 journals. It suggests that paying attention to the papers published in these important journals is helpful to accurately grasp the latest research progress in this field. The number of papers published by institutions can be used to evaluate the influence. Figure 3C lists the top 10 institutions that have published papers, indicating that these institutions have made great contributions to research in this field. Among them, the University of Newcastle and the Hunter Institute of Medicine not only rank in the top 10 in terms of publications, but also have a very close cooperation with each other. In addition, by observing Figure 3D, it is found that Newcastle University is at the core of international cooperation in this field, indicating its important influence in research in this field.

Through the review of the co-occurrence and burst analysis of literature keywords, it is found that researchers mainly focus on the multi-dimensional exploration of the etiology and mechanism of asthmatic airway inflammation, mainly involving the formation mechanism of airway inflammation, the participation of inflammatory cell components and pathway factors. In recent years, some research hot words such as “severe asthma,” “innate lymphoid cells,” “thymic stromal lymphopoietin,” “NF-κB,” “airway remodeling” and “NLRP3 inflammasome” have emerged, and a large number of articles have conducted in-depth research on them. Furthermore, we have conducted in-depth retrospective analysis and discussion of these hot spots. In the process of treating severe asthma, new biological therapies have attracted more and more attention due to the drug tolerance and side effects that traditional glucocorticoids may produce in the treatment of asthma.^[29]^ Biological agents can not only improve clinical symptoms, but also improve some indirect characteristics of airway remodeling.^[30,31]^ Studies have speculated that early use of biological agents in patients with mild or moderate asthma can limit irreversible airway remodeling during the development of severe asthma.^[32]^ Thymic stromal lymphopoietin(TSLP) is located in the key position of the inflammatory response chain, so it is a potential therapeutic target. The treatment strategy of blocking TSLP through a systematic approach has been clinically tested in a wide range of asthma patient populations and has shown positive results.^[33]^ Recent studies have shown that the activity of NLRP3 inflammasome has a significant effect on the development of allergic airway inflammation, which is expected to become a new therapeutic target. It is worth noting that the effect of probiotics on NLRP3 inflammasome provides a new idea for the treatment of asthma.^[34]^ In addition, by analyzing the literature citation, we can gain a deeper understanding of the current progress of asthma airway inflammation. For example, Lambrecht BN et al systematically reviewed and summarized the role of cytokines in airway inflammation of asthma, and predicted that in the next few years, the development of single-cell technology, phenological methods and biotechnology may further unlock the complexity of human asthma, leading to improved animal models and providing improved biotechnology for single-cell factors or multiple cytokine pathways behind specific asthma characteristics.^[35]^ Papi A et al article published in the Lancet in 2018 emphasized the importance of individualized treatment and new treatment strategies in the treatment of asthma.^[36]^ Fahy JV et al further explored the role of type 2 inflammation in asthma and proposed new ideas for studying the disease mechanism independent of type 2 inflammation.^[37]^ It is not difficult to find that the current research mainly focuses on the pathogenesis and therapy of asthmatic airway inflammation. However, the key problem in this field is a continuous research field, so it still needs further research to solve.

### 4.2. Future research trends

From the 2 aspects of keyword burst and citation burst, the future development trend of asthmatic airway inflammation research can be achieved. It was found in Figure 5B that the heat of keywords such as “severe asthma,” “innate lymphoid cells,” “NF-κB,” “airway remodeling,” and “NLRP3 inflammasome” continued to grow. Among several highly popular citations in recent years, the role of cytokines, disease mechanisms, biodiagnostic markers and treatment strategies are the main research contents.^[38]^ It is worth noting that the precise treatment using biological agents is an important research hotspot in the treatment strategy of asthmatic airway inflammation. The above results indicate that in the next few years, international researchers may continue to pay attention to diagnostic markers and biological therapy of asthmatic airway inflammation, and increase investment in related research.

### 4.3. Research advantages and limitations

Through the method of bibliometrics, the existing research results and important research breakthroughs in the field of asthmatic airway inflammation are visualized and analyzed, which points out the future research hotspots and directions for the follow-up researchers. However, this study still has some limitations. First of all, this study only chooses the WOSCC as the data source of the literature, therefore, the literature coverage has certain limitations, which may mean that the articles in other databases are ignored. Secondly, documents in other languages may be ignored because only English documents are included.

## 5. Conclusion

This study systematically describes the current status and breakthrough of research on asthmatic airway inflammation through bibliometrics and visualization analysis methods, and provides important perspectives for future research. Pathogenesis and treatment are the main research directions of asthmatic airway inflammation. The exploration of inflammatory cell components, pathway molecules and biological agents are research hotspots in this field and will remain important research trends in the future. Our study further reviews and summarizes the research hotspots and frontier trends in this field, and it is hoped that this study can provide valuable help for researchers to grasp the current general trend in asthmatic airway inflammation.

## Author contributions

**Conceptualization:** Lang Liye.

**Data curation:** Lang Liye.

**Formal analysis:** Lang Liye.

**Investigation:** Lang Liye.

**Methodology:** Lang Liye, Zhao Hui, Huang Fuchun.

**Validation:** Zhao Hui, Huang Fuchun.

**Visualization:** Lang Liye.

**Writing – original draft:** Lang Liye.

**Writing – review & editing:** Hua Liu.
